# Study protocol: a phase II study of trifluridine/tipiracil plus liposomal irinotecan as second-line therapy in advanced biliary tract cancer (TRITICC 2)

**DOI:** 10.1186/s12885-026-17089-6

**Published:** 2026-10-09

**Authors:** Linde Kehmann, Thorsten Götze, Maria Gonzalez-Carmona, Friedrich Foerster, Marie Berres, Raphael Mohr, Alexander Koch, Samayita Das, Nadja Drusenheimer, Tewodros Kebede, Markus Joerdens, Tom Luedde, Christoph Roderburg

**Affiliations:** 1 Department of Hepatology and Gastroenterology, Charité University Medicine Berlin, Campus Virchow Klinikum, 13353 Berlin, Germany; 2 Servier Deutschland GmbH, 80687 München, Germany; 3 Hospital Northwest, University Cancer Center Frankfurt, 60488 Frankfurt, Germany; 4 The Frankfurt Institute of Clinical Cancer Research IKF GmbH, 60488 Frankfurt, Germany; 5 Department of Internal Medicine I, University Hospital of Bonn, 53127 Bonn, Germany; 6 Department of Internal Medicine I, University Medical Center of the Johannes Gutenberg University Mainz, 55131 Mainz, Germany; 7 Medical Department III, University Hospital of Aachen, 52074 Aachen, Germany; 8 Center for Integrated Oncology - Aachen Bonn Cologne Duesseldorf (CIO-ABCD), 52074 Aachen, Germany; 9 Coordination Center for Clinical Trials, Medical Faculty, University Hospital Duesseldorf, Heinrich Heine – University, 40225 Duesseldorf, Germany; 10 Clinic for Gastroenterology, Hepatology & Infectious Diseases, University Hospital of Düsseldorf, 40225 Düsseldorf, Germany; 11 Center for Integrated Oncology - Aachen Bonn Cologne Duesseldorf (CIO-ABCD), 40225 Duesseldorf, Germany

**Keywords:** Chemotherapy, Biliary tract cancer, Cholangiocarcinoma, FTD/TPI (trifluridine/tipiracil), Gall bladder carcinoma, Nanoliposomal irinotecan, Second-line treatment, Translational research

## Abstract

**Background:**

The prognosis of patients with advanced biliary tract cancer progressing after gemcitabine-based first-line therapy remains poor. Effective second-line treatment options are limited, and outcomes with currently available regimens are modest. Trifluridine/tipiracil (FTD/TPI) and nanoliposomal irinotecan (nal-IRI) have demonstrated clinical activity in gastrointestinal malignancies and may provide synergistic antitumor effects.

**Methods:**

TRITICC-2 is an investigator-initiated, prospective, open-label, multicenter, single-arm phase II trial conducted at academic centers in Germany. The study evaluates the efficacy and safety of FTD/TPI in combination with nal-IRI in patients with histologically confirmed, unresectable or metastatic biliary tract cancer who have progressed on gemcitabine-based first-line therapy. A total of 38 patients are planned for enrollment. Treatment consists of oral FTD/TPI administered on days 1–5 combined with intravenous nal-IRI on day 1 of a 14-day cycle, continued until disease progression, unacceptable toxicity, or withdrawal of consent. The primary endpoint is progression-free survival (PFS). Secondary endpoints include overall survival (OS), objective response rate (ORR), safety, and health-related quality of life. An optional translational research program includes serial liquid biopsies and biomarker analyses. Enrollment is ongoing, with recruitment initiated in late 2025. Efficacy, safety, and translational analyses are pending.

**Conclusion:**

TRITICC-2 investigates a biologically rational combination of FTD/TPI and nal-IRI in a patient population with a high unmet medical need. The study is expected to provide prospective data on the clinical activity and safety of this regimen and may contribute to the identification of biomarkers associated with treatment response in advanced biliary tract cancer.

**Trial registration:**

EUCT 2024-517330-18-00.

**Supplementary Information:**

The online version contains supplementary material available at https://doi.org/10.1186/s12885-026-17089-6.

## Background

Biliary tract cancers (BTC) comprise a biologically and anatomically heterogeneous group of malignancies arising from the intrahepatic and extrahepatic bile ducts as well as the gallbladder and ampulla of Vater. Among these entities, cholangiocarcinoma (CCA) represents the predominant subtype and is classified into intrahepatic (iCCA), perihilar (pCCA), and distal (dCCA) forms according to anatomical location. Although BTCs are considered rare tumors, their incidence has steadily increased over recent decades, particularly for iCCA in Western countries [1, 2]. Globally, BTC accounts for approximately 3% of all gastrointestinal malignancies and represents the second most common primary hepatic cancer after hepatocellular carcinoma [1, 2]. In Germany alone, approximately 5,000 new cases are diagnosed annually [3, 4]. Due to the absence of specific early symptoms, most patients present with locally advanced or metastatic disease at initial diagnosis, precluding curative resection in the majority of cases and contributing to the persistently poor overall prognosis [1, 2].

For more than a decade, systemic therapy for advanced BTC was defined by the results of the pivotal ABC-02 trial, which established the combination of gemcitabine and cisplatin (GemCis) as first-line standard of care [5]. Compared with gemcitabine monotherapy, GemCis significantly improved overall survival (OS) and progression-free survival (PFS), with a median OS of 11.7 months compared to 7.9 months under Gemcitabine [5]. More recently, the integration of immune checkpoint inhibition has reshaped the first-line therapeutic landscape. The addition of the PD-L1 inhibitor durvalumab to GemCis in the TOPAZ-1 trial resulted in a statistically significant and clinically meaningful improvement in OS and PFS compared with chemotherapy alone.

(12,8 months vs. 11,5 months and 7,2 months vs. 5,7 months, respectively; [6]). Similar findings were reported in the KEYNOTE-966 study evaluating pembrolizumab in combination with GemCis [7]. Consequently, chemo-immunotherapy has become the current first-line standard in patients with advanced BTC and preserved performance status. Despite these advances, disease progression under first-line therapy remains inevitable in the majority of patients. Importantly, only approximately 15–25% of patients are eligible for second-line systemic treatment due to rapid clinical deterioration [8, 9]. For those who maintain sufficient functional status, therapeutic options remain limited and the magnitude of benefit modest. The ABC-06 phase III trial demonstrated a statistically significant but clinically moderate survival advantage for modified FOLFOX (5-Fluoruracil, Leucovorin (LV) and Oxaliplatin) compared with active symptom control, establishing fluoropyrimidine-based combination chemotherapy as a reference second-line regimen [10]. However, objective response rates were low and median OS remained below seven months, underscoring the urgent need for improved treatment strategies.

Irinotecan-based regimens have emerged as an alternative approach in the second-line setting. Phase II data suggest that fluoropyrimidine–irinotecan combinations such as FOLFIRI provide disease control in selected patients [11]. More recently, nal-IRI, a liposomal formulation designed to prolong systemic exposure and enhance intratumoral drug delivery, has demonstrated clinically relevant activity in BTC. In the randomized phase IIb NIFTY trial, the addition of nal-IRI to fluorouracil and leucovorin significantly prolonged PFS and OS compared with fluorouracil and leucovorin alone in patients previously treated with GemCis [12]. While the subsequent European NALIRICC trial did not reproduce the magnitude of benefit observed in NIFTY [13], pooled analyses suggest that irinotecan-based doublets may offer meaningful benefit in molecularly unselected patient populations when carefully selected [14]. Nevertheless, outcomes remain suboptimal and there is no universally accepted global standard beyond fluoropyrimidine-based chemotherapy. Parallel to advances in cytotoxic therapy, comprehensive molecular profiling has revealed a diverse genomic landscape in BTC. Targetable alterations such as IDH1 mutations, FGFR2 fusions, HER2 amplifications, BRAF mutations, and MSI-H/dMMR status have led to the development of precision oncology approaches [15–18]. However, these alterations are restricted to specific subgroups—predominantly intrahepatic tumors—and collectively apply to only a subset of patients. The majority of individuals with advanced BTC lack actionable genomic alterations and remain dependent on conventional chemotherapy strategies. Therefore, optimizing cytotoxic backbone regimens continues to represent a critical clinical objective.

FTD/TPI is an orally administered nucleoside analogue consisting of trifluridine, which is incorporated directly into DNA leading to DNA dysfunction, and tipiracil, a thymidine phosphorylase inhibitor that increases systemic bioavailability of trifluridine [19]. Unlike 5-fluorouracil (5-FU), trifluridine exerts its primary cytotoxic effect through DNA incorporation rather than thymidylate synthase inhibition, thereby partially overcoming mechanisms of fluoropyrimidine resistance [20]. FTD/TPI has demonstrated survival benefit in refractory metastatic colorectal cancer (mCRC) in the RECOURSE trial and in advanced gastric cancer in the TAGS trial, leading to regulatory approval in these indications [21, 22]. Importantly, clinical activity has been observed in tumors previously exposed to 5-FU, supporting its potential utility in BTC patients pretreated with fluoropyrimidine-containing regimens. Preclinical and early clinical data suggest synergistic activity between FTD/TPI and irinotecan. The combination has shown tolerable safety profiles and encouraging antitumor activity in gastrointestinal malignancies, including preliminary signals in advanced BTC [23–25]. Mechanistically, irinotecan-induced DNA strand breaks may enhance incorporation of trifluridine into DNA, potentially augmenting cytotoxic efficacy [26]. Furthermore, the use of nal-IRI may improve pharmacokinetic stability and tumor exposure compared with conventional irinotecan formulations, providing a biologically plausible rationale for combination strategies.

Taken together, despite incremental improvements in first-line chemo-immunotherapy and the emergence of biomarker-driven approaches, the prognosis of patients with advanced BTC progressing after gemcitabine-based therapy remains poor. The limited efficacy of currently available second-line regimens highlights the need for innovative combination strategies that can be administered safely in a population with compromised performance status. The combination of FTD/TPI and nal-IRI represents a rational therapeutic approach aiming to enhance antitumor activity through complementary mechanisms of action while maintaining manageable toxicity. The TRITICC-2 study was therefore initiated to prospectively evaluate the efficacy, safety, and translational correlates of this regimen in patients with advanced biliary tract cancer following progression on gemcitabine-based first-line therapy.

## Objectives and endpoints

### Objectives

The primary objective of the TRITICC-2 trial is to examine the efficacy of a combination therapy of FTD/TPI and nal-IRI in patients with advanced, non resectable or metastatic cholangio- and gallbladder carcinoma after failure to respond to a previous gemcitabine-based treatment. Secondary objectives aim to determine safety, quality of life and further efficacy parameters in the study participants.

### Endpoints

The primary endpoint is median progression free survival (PFS) assessed by the local investigator at each site (time from first administration of chemotherapy to the date of radiological or clinical tumor progression or death of any cause whichever comes first). Secondary endpoints are PFS rate at 4 months, median overall survival and proportion of patients with an objective response according to RECIST 1.1. Secondary safety endpoints are type, grade, frequency and severity of adverse events according NCI CTCAE version 5.0. Secondary endpoint quality of life will be assessed by EORTC QLQ-30 and the EQ-5D-5Lquestionnaires. Secondary translational endpoints are cfDNA exome sequencing, circulating miRNAs and lncRNAs, concentrations of inflammatory cytokines and gut microbiome analysis.

The study protocol is summarized in Table 1.

**Table 1:** Objectives and endpoints of the TRITICC-2 trial

• **Objectives**
**Primary Objective**
To examine the efficacy of a combination therapy of FTD/TPI and nal-IRI in patients with advanced, non resectable or metastatic cholangio- and gallbladder carcinoma after failure to respond to a previous gemcitabine-based treatment.
**Secondary Objectives**
• To determine efficacy and safety of the treatment concept
• To determine health-related quality of life (HR-QoL)
• To determine translational observations (circulating miRNAs and lncRNAs, concentrations of inflammatory cytokines, gut microbiome analysis)
• **Endpoints**
**Primary Endpoint**
• Median progression free survival (PFS) assessed by the local investigator
**Secondary Endpoints**
• Progression-free survival rate at 4 months
• Median overall survival
• Proportion of patients with an objective response according to RECIST 1.1
• Type, frequency and severity of adverse events with severity according to NCI CTCAE version 5.0
o **Health-Related Quality of Life**
• HR-QoL according to EORTC QLQ-C30 and the EQ-5D-5 L
• **Endpoints optional translational research**
• Analysis of concentrations of **circulating tumor cells** in the patient’s sera and correlations with PFS and response to therapy.
• Analysis of extracellular vesicle size distribution and concentrations, measured by **nanoparticle tracking analysis (NTA)** in patients´ sera. Correlation of the baseline composition and alterations during therapy with PFS and ORR.
• **cfDNA exome sequencing**: a panel of 28 mutations (e.g. IDH1, IDH2, SMAD4) that are specific for CCA will be recapitulated in patient’s serum by using cfDNA exome sequencing.
• **Circulating miRNAs and lncRNAs**: Alterations in serum miRNAs as prognostic markers will be analysed using array and qPCR- based techniques.
• **Concentrations of inflammatory cytokines**: A panel of inflammatory cytokines will be analyzed using FACS bead technology.
• **Gut microbiome analysis**: By using high-throughput sequencing of bacterial 16 S RNA a complete and unbiased analysis of the gut microbiota will be performed in each individual patient. Both, the baseline composition and alterations during therapy will be correlated with PFS and response to therapy.

## Methods/study design

TRITICC-2 is an investigator-initiated, prospective, open-label, multicentre, single-arm phase IIa clinical trial sponsored and coordinated by the Heinrich-Heine-University Duesseldorf. The study evaluates systemic therapy with FTD/TPI in combination with nal-IRI in patients with histologically or cytologically confirmed, unresectable locally advanced or metastatic cholangiocarcinoma or gallbladder carcinoma. Eligible patients who have experienced disease progression following gemcitabine-based first-line chemotherapy receive the study treatment in 14-day cycles. A total of 38 patients, including an allowance for three anticipated dropouts or non-evaluable cases, are planned for enrolment across up to six participating tertiary care centres. It is estimated that approximately 45 patients will need to be screened to achieve the target sample size.

### Study duration

Patient recruitment commenced in the last quarter of 2025. The recruitment period will last 24 months, treatment duration is estimated to be around 4 months on average followed by a 6 months follow-up period and data cleaning, processing, analysis and reporting 12 months. Based on these assumptions, duration will be in total 46 months including evaluation and clinical study report.

### Trial population

Patients with biliary tract cancer after progressing on a gemcitabine based first-line therapy and fulfilling the other inclusion criteria will be eligible to participate in this clinical trial.

### Inclusion criteria

Subjects, fulfilling the following inclusion criteria are suitable for participation in the study:

1. Written informed consent and any locally-required authorization (EU Data Privacy Directive in the EU) prior to performing any protocol-related procedures, including screening evaluations.
2. Age ≥ 18 years at time of study entry.
3. Histologically or cytologically confirmed, non-resectable, locally advanced or metastatic cholangiocarcinoma or gallbladder carcinoma.
4. Patients with available molecular tumor profiling may only be included if they are not eligible for approved targeted therapies or if they reject approved targeted therapies. Eligibility for such therapies is determined by the local interdisciplinary tumor board or molecular tumor board, based on the results of molecular testing, clinical criteria, and the patient’s preference.
5. Measurable or assessable disease according to RECIST 1.1.
6. Documented disease progression after prior gemcitabine or gemcitabine-containing therapy or intolerance to such a treatment. Examples of permitted therapies include, but are not limited to: (a) Single agent gemcitabine); (b) Any gemcitabine-based regimen, with or without maintenance gemcitabine.
7. ECOG performance status 0–1.
8. Ability to take medications orally.
9. Adequate blood count, liver-enzymes, and renal function as follows:

- ANC ≥ 1,500 cells/µL and Platelet count of ≥ 100 × 10^9^/L (≥ 100,000 per mm^3^) and Hemoglobin ≥ 8 g/dL (blood transfusions are permitted for patients with hemoglobin levels below 9 g/dL).
- Serum total bilirubin of ≤ 1.5x upper normal limit (ULN) (biliary drainage is allowed for biliary obstruction; elevated bilirubin should be caused by obstruction not impaired liver function as assessed by albumin and INR values).
- Albumin levels ≥ 2.8 g/dL.
- Patients not receiving therapeutic anticoagulation must have an INR < 1.5 ULN and PTT < 1.5 ULN within 7 days prior to inclusion. The use of full dose anticoagulants is allowed as long as the INR or PTT is within therapeutic limits (according to the medical standard in the institution) and the patient has been on a stable dose for anticoagulants for at least three weeks at the time of inclusion.
- AST (SGOT) and ALT (SGPT) ≤ 5x institutional upper limit of normal.
- Serum Creatinine ≤ 1.5 x ULN and a calculated glomerular filtration rate ≥ 30 mL per minute.

10. Women of childbearing potential must have a negative pregnancy test and must agree to adequate birth control. Males must agree to adequate birth control.

### Exclusion criteria

Subjects, fulfilling one or more of the following exclusion criteria will not be included in the study:

1. Age < 18 years.
2. CNS metastases.
3. Active, uncontrolled infection.
4. Additional malignancy within the past 2 years (except adequately treated in-situ carcinoma of the cervix or non-melanoma skin cancer).
5. Clinically significant gastrointestinal disorders including bleeding, inflammation, occlusion, or diarrhoea > grade 1.
6. Any condition or comorbidity that, in the opinion of the investigator, would interfere with evaluation of study treatment or interpretation of patient safety or study results.
7. Known hypersensitivity FTD/TPI or Nal-IRI or their components.
8. Medication that is known to interfere with any of the agents applied in the trial.
9. Pregnant or lactating female.
10. Prior total gastrectomy.
11. Previous radio- or radiochemotherapy, previous transarterial chemoembolisation (TACE), radiofrequency ablation (RFA) or selective intraarterial radiotherapy (SIRT) within 3 months prior to inclusion (except radiation for bone metastases).
12. Patients who might be dependent on the sponsor, site or the investigator.
13. Persons held in an institution by legal or official order.
14. Liver cirrhosis, except Child-Pugh A.
15. Patients who are unable to consent because they do not understand the nature, significance and implications of the clinical trial and therefore cannot form a rational intention in the light of the facts.
16. Participation in another study with ongoing use of unlicensed investigational product from 28 days or < 5 half-lives of the investigational product before study enrollment.

### Gender and age selection

Adults of all genders equal or above 18 years are eligible for this clinical trial.

### Tumor imaging

Tumor response and disease progression will be evaluated in accordance with RECIST version 1.1 criteria. Whenever feasible, magnetic resonance imaging (MRI) should be used as the preferred imaging modality for tumor assessment. Alternatively, contrast-enhanced computed tomography (CT) is acceptable. The initially selected imaging technique must be used consistently for all subsequent tumor evaluations throughout the study to ensure comparability. Thoracic imaging by CT is required for comprehensive staging. In cases of suspected bone metastases, additional diagnostic procedures such as skeletal scintigraphy and/or targeted radiographs of affected skeletal regions should be performed. If central nervous system (CNS) involvement is clinically suspected, cranial imaging is mandatory, with MRI being the preferred modality; cranial CT may be used if MRI is not feasible. Further imaging investigations may be conducted in patients presenting with new or worsening symptoms, if deemed clinically necessary by the investigator.

### Treatment regimen

Patients will receive FTD/TPI (Lonsurf^®^) and nal-IRI (Onivyde^®^) until radiological disease progression or inacceptable toxicity. Dosage and schedule were selected according to the results of the “Phase 1 study of nal-IRI in combination with FTD/TPI in refractory solid tumors” (Clinical trial information: NCT03810742), Table 2:

- FTD/TPI (Lonsurf^®^) will be administered at a dose of 30 mg/m^2^ / dose twice daily on days 1–5 in14-day cycles.
- nal-IRI (Onivyde^®^) will be administered at the same day as FTD/TPI (Lonsurf^®^) on day 1 of each 14-day cycle at a dose of 70 mg/m^2^ / dose.

**Table 2:** Calculation of dosing for FTD/TPI in mg according to BSA used in the TRITICC-2 trial

dose	BSA (m^2^)	Dose in mg (2x daily)	Tablets per dose (2x daily)		Total daily dose (mg)
			15 mg/6.14 mg	20 mg/8.19 mg	
30 mg/m^2^	< 1.09	30 mg	2	0	60
	1.09–1.24	35 mg	1	1	70
	1.25–1.39	40 mg	0	2	80
	1.40–1.54	45 mg	3	0	90
	1.55–1.69	50 mg	2	1	100
	1.70–1.94	55 mg	1	2	110
	1.95–2.09	60 mg	0	3	120
	2.10–2.28	65 mg	3	1	130
	≥ 2.29	70 mg	2	2	140

#### Dose modifications and dose delays

Toxicity is classified according to NCI-CTCAE version 5.0; the dose modifications are made based on the grading given in the latter. Doses will be reduced for haematological and non-haematological toxicities. In case of concurrent toxicities, all dose modifications should be based on the worst preceding toxicity. FTD/TPI and nal-IRI doses must be reduced according to recommendations below. Patients who require further dose reduction should discontinue treatment. Reescalation of dose levels is not permitted. If any toxicity is unequivocally caused by only one drug, a dose modification of the other drug is not required. If any toxicity is unequivocally caused by nal-IRI and the dose modification require the discontinuation of nal-IRI, it is allowed to continue treatment with FTD/TPI according to the decision of the local investigator who should contact the Coordinating Investigator.

#### Dose modification of FTD/TPI

A maximum of 2 dose reduction level (25 mg/m^2^ BSA BID; 20 mg/m^2^ BSA BID) is permitted for FTD/TPI. Tables 3 and 4 display the dose in mg to be taken twice daily calculated per m^2^ BSA. Tables 5 and 6 display the dose interruption, resumption and reduction criteria for haematological and non-haematological adverse reaction.

**Table 3:** Calculation of dose reduction to 25 mg/m^2^ for FTD/TPI in mg according to BSA used in the TRITICC-2 trial

dose	BSA (m^2^)	Dose in mg (2x daily)	Tablets per dose (2x daily)		Total daily dose (mg)
			15 mg/6.14 mg	20 mg/8.19 mg	
25 mg/m^2^	< 1.10	25 mg!	2!	1!	50!
	1.10–1.29	30 mg	2	0	60
	1.30–1.49	35 mg	1	1	70
	1.50–1.69	40 mg	0	2	80
	1.70–1.89	45 mg	3	0	90
	1.90–2.09	50 mg	2	1	100
	2.10–2.29	55 mg	1	2	110
	≥ 2.30	60 mg	0	3	120

(!) At a total daily dose of 50 mg, patients should take 1 × 20 mg tablet in the morning and 2 × 15 mg tablets in the evening

**Table 4:** Calculation of dose reduction to 20 mg/m^2^ for FTD/TPI in mg according to BSA used in the TRITICC-2 trial

dose	BSA (m^2^)	Dose in mg (2x daily)	Tablets per dose (2x daily)		Total daily dose (mg)
			15 mg/6.14 mg	20 mg/8.19 mg	
20 mg/m^2^	< 1.14	20 mg	0	1	40
	1.14–1.34	25 mg!	2!	1!	50!
	1.35–1.59	33 mg	2	0	60
	1.60–1.94	35 mg	1	1	70
	1.95–2.09	40 mg	0	2	80
	2.10–2.34	45 mg	3	0	90
	≥ 2.35	50 mg	2	1	100

(!) At a total daily dose of 50 mg, patients should take 1 × 20 mg tablet in the morning and 2 × 15 mg tablets in the evening

**Table 5:** Dose interruption and resumption criteria for FTD/TPI for haematological toxicities related to myelosuppression

Parameter	Interruption	Resumption criteria^a^
Neutrophils	< 0.5 × 10^9^/L	≥ 1.5 × 10^9^/L
Platelets	< 50 × 10^9^/L	≥ 100 × 10^9^/L

^a^ Resumption criteria applied to the start of the next cycle for all patients regardless of whether or not the interruption criteria were met

**Table 6:** Recommended dose modifications for FTF/TPI in case of haematological and non-haematological adverse reactions

Adverse reaction	Recommended dose modifications
• Febrile neutropenia	Interrupt dosing until toxicity resolves to Grade 1 or baseline. • When resuming dosing, decrease the dose level by 5 mg/m^2^/dose from theprevious dose level • Dose reductions are permitted to a minimum dose of 20 mg/m^2^/doseBID daily.
• Grade 4 neutropenia (< 0.5 × 10^9^/L) or thrombocytopenia (< 25 × 10^9^/L) that results in more than 1 week’s delay in start of next cycle	
• non-haematologic Grade 3 or Grade 4 adverse reaction; except for Grade 3 nausea and/or vomiting controlled by antiemetic therapy or diarrhoea responsive to antidiarrhoeal medicinal products	

#### Dose modification of nal-IRI

Table 7 displays recommended dose modifications for nal-IRI in case of haematological or non-haematological adverse reactions.

**Table 7:** Recommended dose interruption/ modification criteria for nal-IRI

Toxicity grade (value) by NCI CTCAE	Nal-IRI adjustment	
**Haematological toxicities**		
**Neutropenia**	A new cycle of therapy should not begin until the absolute neutrophil count is ≥ 1,500 cells/mm^3^	
*Grade 3 or Grade 4 (< 1*,*000 cells/mm*^*3*^*)* or Neutropenic fever	*First occurrence*	Reduce Nal-IRI dose to 50 mg/m^2^
	*Second occurrence*	Reduce Nal-IRI dose to 43 mg/m^2^
	*Third occurrence*	Discontinue treatment
**Thrombocytopenia** **Leukopenia**	A new cycle of therapy should not begin until the platelet count is ≥ 100,000 platelets/mm^3^ Dose modifications for leukopenia and thrombocytopenia are based on NCI CTCAE toxicity grading and are the same as recommended for neutropenia above.	
**Non-haematological toxicities**		
**Diarrhoea**	A new cycle of therapy should not begin until diarrhoea resolves to ≤ Grade 1 (2–3 stools/day more than pre-treatment frequency).	
*Grade 2*	A new cycle of therapy should not begin until diarrhoea resolves to ≤ Grade 1 (2–3 stools/day more than pre-treatment frequency).	
*Grade 3 or 4*	*First occurrence*	Reduce Nal-IRI dose to 50 mg/m^2^
	*Second occurrence*	Reduce Nal-IRI dose to 43 mg/m^2^
	*Third occurrence*	Discontinue treatment
**Nausea/vomiting**	A new cycle of therapy should not begin until nausea/vomiting resolves to ≤ Grade 1 or baseline	
*Grade 3 or 4 (despite antiemetic therapy)*	*First occurrence*	Optimise antiemetic therapy Reduce Nal-IRI dose to 50 mg/m^2^
	*Second occurrence*	Optimise antiemetic therapy Reduce Nal-IRI dose to 43 mg/m^2^
	*Third occurrence*	Discontinue treatment
**Hepatic**,** renal**,** respiratory or other2 toxicities**	A new cycle of therapy should not begin until the adverse reaction resolves to ≤ Grade 1	
*Grade 3 or 4*	*First occurrence*	Reduce Nal-IRI dose to 50 mg/m^2^
	*Second occurrence*	Reduce Nal-IRI dose to 43 mg/m^2^
	*Third occurrence*	Discontinue treatment
**Anaphylactic reaction**	*First occurrence*	Discontinue treatment
**Interstitial Lung Disease**	*First occurrence*	Discontinue treatment

### Prohibited concomitant medication

The following medications/treatments are not allowed during the study participation, unless the patient is in the follow-up phase and has permanently discontinued any study medication: Any investigational medicinal product or experimental therapy or participation in another clinical trial. Any other anti-tumor treatment including chemotherapy, targeted therapy, antibodies, any antihormonal tumor treatment, and immunotherapy other than FTD/TPI and nal-IRI.

### Permitted concomitant medication

All medication, which is considered necessary for the patient’s welfare, and which is not expected to interfere with the evaluation of FTD/TPI or nal-IRI, may be given at the discretion of the investigator. All concomitant medications must be recorded in the patient’s source documentation, as well as in the appropriate pages of the eCRF. Subjects should receive full supportive care, including transfusions of blood and blood products, anti-infective drugs, and antiemetics when appropriate. All patients should be closely monitored for side effects of all concomitant medications regardless of the path of elimination.

#### Antiemetics

During the treatment with FTD/TPI and nal-IRI, owing a moderate emetic potential, an antiemetic treatment should be administered according to center-specific protocols using, among others, dexamethasone and 5-HT3 antagonists. In case of insufficient effect, aprepitant (Emend^®^) may be considered. Appropriate prophylactic antiemesis is recommended.

#### Anticoagulants

The use of full dose anticoagulants is allowed as long as the INR or PTT is within therapeutic limits (according to the medical standards in the institution). During treatment close monitoring of INR for oral anticoagulants is recommended.

#### Growth factors

Hematopoietic growth factors (i.e., G-CSF) may be used according to institutional guidelines to treat febrile neutropenia.

For comprehensive information on contraindications and precautions, investigators are advised to consult the current Summary of Product Characteristics (SmPC). Caution is warranted when co-administering medicinal products that are substrates of human thymidine kinase, such as zidovudine. When given together with FTD/TPI, these agents may compete with trifluridine for intracellular activation via thymidine kinases, potentially reducing the antiviral efficacy. In patients requiring antiviral therapy with thymidine kinase substrates, careful monitoring for diminished antiviral response is recommended. If appropriate, consideration should be given to switching to an alternative antiviral agent that is not dependent on human thymidine kinase for activation, such as lamivudine or abacavir. It is currently unknown whether FTD/TPI may impair the effectiveness of hormonal contraceptives. Consequently, women of childbearing potential using hormonal contraceptive methods should additionally employ a reliable barrier method of contraception during treatment.

### Statistical considerations

The full analysis population will include all participants who were enrolled in the study and have received at least one dose of study medication. The intention-to-treat (ITT) population will include all participants who were enrolled, regardless of whether they adhered to the treatment. Baseline and demographic variables will be presented with descriptive statistics (means and SDs, medians or frequencies (percentages)) in the intention-to-treat population. The primary outcome variable will be analysed based on the intention-to-treat population. The secondary outcome variables will be analysed in the full analysis population. The primary and secondary endpoint variables will be described using frequencies and percentages, and the minimum, the mean, the median, the interquartile range (IQR), the maximum and the number of missing observations for each relevant variable. Adverse events will be tabulated in listings for each study participant. The primary endpoint, PFS, will be analysed using the Kaplan-Meier method to estimate the survival function over time. To this end the method of Turnbull will be used to compute the survival curve (Turnbull, B. W. (1976). J R Stat Soc Series B 38:290–295.). The Turnbull estimator is an extension of the Kaplan-Meier estimator that can handle interval-censored data, where the exact event time is not known but falls within a certain interval. This is useful in scenarios where subjects are only periodically monitored. The variance of the survival will be estimated using the Greenwood formula. The log transformation will be used for the calculation of confidence intervals. To explore the relationship between covariates and survival outcomes, the cox proportional hazards regression model will be fitted if the proportional hazard assumption is met. An exploratory analysis of secondary outcomes will be performed. Overall survival will be analysed using the Kaplan-Meier estimate of survival. If proportional hazards assumption is met, the cox proportional hazards regression model will be applied to investigate the relationship between covariates and overall survival. For the secondary outcomes, such as objective response and summary of the occurrence of adverse events will be computed descriptively. For objective response data, confidence intervals for proportions will be calculated using the Wilson method (Newcombe RG. Stat. Med. 1998;17:857–872). Adverse Events (AEs) will be categorized according to their severity grades and summarized in a tabular format. The severity of each AE will be classified using the Common Terminology Criteria for Adverse Events (CTCAE), ranging from mild (Grade 1) to life-threatening (Grade 4) or death (Grade 5). The summary table will present the number and percentage of patients experiencing each severity grade. For quality-of-life data, linear mixed effect models will be used considering time as a fixed effect and individual patients as a random effect. If missing data occurred more than anticipated, an appropriate imputation method will be used considering missing completely at random (MCAR) or missing at random (MAR) missingness mechanisms. The complete statistical analysis methods will be described in the first version of the statistical analysis plan (SAP).

#### Sample size determination

A total number of 31 events produces a two-sided 95% confidence interval with a width equal to 2.244 when the estimate of the median survival time is 3 months. This gives a confidence interval with a lower limit of 2.172 and an upper limit of 4.415 for the median survival time. The censoring probability is expected to be 10% giving an estimated sample size of 35, assuming Type-II censoring where the study is run until targeted number of events occur. Assuming a ~ 10% drop out rate the final sample size is calculated as 38.

### Assessment of adverse events

The severity of all adverse events (AEs) and serious adverse events (SAEs) will be graded according to the National Cancer Institute Common Terminology Criteria for Adverse Events (NCI-CTCAE), version 5.0. All study personnel involved in trial conduct must have access to the current NCI-CTCAE v5.0 documentation. Each adverse event must be recorded with comprehensive details including: the clinical diagnosis or description of the event, duration, severity grade, classification as serious or non-serious (events meeting seriousness criteria require additional SAE reporting), assessment of causality in relation to the investigational medicinal products, and the final outcome of the event. All adverse events occurring from the time the participant signs informed consent until 30 days after the end of treatment visit must be documented. A serious adverse event (SAE) or serious adverse drug reaction (SADR) is defined as any adverse event that meets at least one of the following criteria: results in death; is life-threatening; necessitates inpatient hospitalization or prolongation of existing hospitalization; results in persistent or significant disability/incapacity (a substantial disruption of the subject’s ability to conduct normal life functions); results in a congenital anomaly or birth defect; or represents another medically important event. Medically important events are those that may not be immediately life-threatening or result in death or hospitalization but may place the patient at risk or require medical or surgical intervention to prevent one of the outcomes listed above. Determination of seriousness requires appropriate medical and scientific judgment. All SAEs must be reported within 24 h after the investigator becomes aware of the event.

### Translational research

Plasma and serum samples will be collected from all participants who provide separate written informed consent for translational research. Sampling will be performed at baseline, at each scheduled restaging assessment, and at the end-of-treatment visit. Following centrifugation, serum and plasma aliquots will be stored at − 80 °C until further analysis. Inflammatory cytokine concentrations will be quantified in serum using fluorescence-activated cell sorting (FACS) bead-based technology. The predefined panel includes Osteopontin, suPAR, APRIL, TWEAK, TRAIL, CCL1, CCL2, CCL5, and CXCR1, CXCR2, CXCR5, and CXCR9, all of which have been reported to be altered in the circulation of patients with gastrointestinal malignancies. In addition, circulating microRNAs (miRNAs) and long non-coding RNAs (lncRNAs) will be measured in serum samples. These retrospective biomarker analyses aim to explore potential predictive associations with treatment response, survival outcomes, and mechanisms of resistance. For translational investigations involving circulating tumor-derived material, liquid biopsy samples will be obtained after separate informed consent. Cell-free DNA (cfDNA) extracted from plasma will undergo exome sequencing using a targeted panel comprising 28 mutations (including, for example, IDH1, IDH2, SMAD4, among others). Detected genomic alterations will be correlated with clinical endpoints such as treatment response, overall survival, and development of resistance. Stool samples will also be collected at baseline, at each restaging time point, and at treatment discontinuation following separate informed consent. To characterize the gut microbiota, molecular genetic analyses will be performed. Stool-derived DNA will be isolated and bacterial 16 S rRNA gene sequences will be quantified. Initial screening will be conducted using quantitative PCR with group-specific primers targeting variable regions of bacterial 16 S rRNA genes. Subsequently, a defined set of 15 primer pairs directed against predominant gut bacterial species will be applied to assess rifaximin-associated changes. In addition, high-throughput sequencing of bacterial 16 S rRNA genes will be performed to enable comprehensive and unbiased profiling of the intestinal microbiome.

### QoL assessment

Quality of life (QoL) will be evaluated using the EORTC QLQ-C30 (version 3.0) and the EQ-5D-5 L questionnaires. The EORTC QLQ-C30 is a validated instrument for assessing patient-reported health-related quality of life in oncology. It comprises 30 items organized into nine multi-item scales, including five functional domains (physical, role, cognitive, emotional, and social functioning), three symptom domains (fatigue, pain, nausea/vomiting), and one global health status/QoL scale. In addition, several single-item symptom measures are included, covering dyspnoea, insomnia, appetite loss, constipation, diarrhoea, and financial impact related to disease or treatment. The EQ-5D-5 L, developed by the EuroQol Group, is a standardized instrument for measuring health status. It consists of two components: the descriptive system and the EQ visual analogue scale (EQ VAS). The descriptive system assesses five dimensions—mobility, self-care, usual activities, pain/discomfort, and anxiety/depression—each with five response levels (no problems, slight problems, moderate problems, severe problems, extreme problems). Patients select one response per dimension, resulting in a five-digit health state profile. These numeric levels represent categories only and do not constitute arithmetic values. The EQ VAS captures the patient’s self-rated health on a 20-cm vertical scale ranging from “the best health you can imagine” to “the worst health you can imagine.” Patients are instructed to indicate their current health status by marking an “X” on the scale and to record the corresponding numeric value. EQ VAS results will be summarized using mean values and standard deviations, while EQ-5D-5 L descriptive results will be presented as health profiles and index scores. Paper versions of the EORTC QLQ-C30 and EQ-5D-5 L questionnaires will be provided to patients on Day 1 of Cycle 1 prior to any other study-related procedures. Thereafter, questionnaires will be completed at each restaging visit, the end of treatment visit and the two follow-up visits.

### Ethical considerations and trial registration

This trial will be conducted in accordance with the current ICH-GCP-guidelines. Good Clinical Practice (ICH-GCP E6 (R3)) is an international ethical and scientific quality standard for designing, conducting, recording and reporting trials that involve the participation of human subjects. Compliance with this standard provides public assurance that the rights, safety and well-being of trial subjects are protected, consistent with the principles that have their origin in the Declaration of Helsinki, and that the clinical trial data are credible.

Any change in the study protocol and/or informed consent form will be presented to the member state concerned and approval will be obtained before implementation of the changes. The sponsor, the competent authorities and the ethics committee may stop the clinical trial or participation of a clinical trial site in the clinical trial for medical, safety, regulatory, administrative or other reasons consistent with ICH-GCP, the respective European Union’s and national legislation. The study is registered at the Clinical Trial Information System (CTIS) (EUCT 2024-517330-18-00).

### Summary of study procedures

**Table:** 

Study Period	SCR phase	Treatment Period		EOT	FU
Visit No	1	2 - X		X	(X + 1) – (X + 2)
Activity	Screening	Investigation	Restaging	End of Treatment	Follow Up
**Time**	**within 28 days before treatment**	**day 1 of each 14-day cycle (before adminis-tration)**	**each 8 weeks after start of treatment**	**when disease progression is determined**	**every 3 months after EOT up to 6 months**
**Visit window +/- calendar days**	**− 28 days**	**− 48 h**	**± 1 week**	**+ 1 week**	**± 1 weeks**
**Informed consent**	**X**				
**Inclusion and exclusion criteria**	**X**				
**Pregnancy test in FCBPs**	**X**	X***		X	X
**Demographics**	**X**				
**Medical history**	**X**				
**12 channel-ECG**	**X**				
**Echocardiography**	**X**				
**Administration of study drug** ^1^		------------			
**Tumor assessment according to RECIST version 1.1** ^2^	**X**		**X**	X**	
**Tumor markers** - CEA and CA19-9	**X**		**X**	X*	
**Concomitant medication** (continuously)	------------------------				
**Physical examination** - height, weight, pulse and blood pressure	**X**	**X**		**X**	
**ECOG performance status**	**X**	**X**		**X**	
**Blood and urine analysis** ^3^	**X**	**X**		**X**	
**Life quality assessment** EORTC QLQ-C30 and EQ-5D-5Lquestionnaires	**X**		**X**	X*	**X**
***EDTA blood (12 ml;*** ***optional*** ***)***	**X**		**X**	X*	
***Plasma and serum samples (2 × 10 ml;*** ***optiona*** ***l)***	**X**		**X**	X*	
***Liquid biopsy*** ***(optional)*** *− 20 ml blood in Streck tubes*	**X**		**X**	X*	
***Stool collection*** ***(optional)*** *- in Stratec stool collection tubes*	**X**		**X**	X*	
**AE evaluation** ^4,5^	---------------------------------				
**Documentation of the subsequent treatments for CCA**				**X**	**X**
**Documentation of progression and survival data**		**X**	**X**	X*	**X**

^1^Trifluridin/tipiracil will be administered at a dose of 30 mg/m2 / dose twice daily on days 1-5 followed by a 9-day recovery period from day 6 trough day 14 of each 14-day treatment cycle. Nanoliposomal irinotecan will be administered at the same time as trifluridin/tipiracil on day 1 of each cycle at a dose of 70 mg/m2 / dose

^2^See Chapter 5.6.1 for details of imaging examinations for tumor assessment according to RECIST version 1.1. Whenever possible, MRI should be chosen as examination method. However, conventional CT is permitted as alternative. The examination method once selected is to be retained for all examinations within the context of the study. The initial CT/MRI showing progress under first-line therapy for inclusion should not been older than 28 days calculated from the time point of inclusion

^3^Including leukocytes, neutrophil granulocytes, lymphocytes, thrombocytes, erythrocytes, haemoglobin, Creatinine, creatinine clearance according to Cockcroft and Gault, urea, LDH, total bilirubin, ALAT, AST, alkaline phosphatase, sodium, potassium, calcium, magnesium, INR, serum albumin. Urine dipstick analysis to monitor proteinuria.^*^Only if EOT is performed more than 2 weeks after restaging showing PD^**^Only if EOT is performed without previous restaging showing PD or if restaging was more than 8 weeks ago^***^Before start of every other cycle

^4^According to NCI CTCAE version 5.0 (starting with the date of signed informed consent)

^5^All AEs will be recorded by the Investigator in the subject’s source document and in the eCRF from the time of signed informed consent by the patient until 30 days after the end of treatment visit. Moreover, those AEs made known to the investigator at any time thereafter that are suspected of being related to IMP and those that result in death independent of a causal relationship to the IMP have to be recorded^*^Only if EOT is performed more than 2 weeks after restaging showing PD

## Discussion

Biliary tract cancers (BTC) remain associated with a poor prognosis, particularly after progression on gemcitabine-based first-line therapy. Although the addition of immune checkpoint inhibitors to chemotherapy has modestly improved outcomes in the first-line setting [6, 7], the majority of patients ultimately experience disease progression and require further treatment. In this setting, therapeutic options are limited and associated with only modest clinical benefit. The ABC-06 trial established modified FOLFOX as a reference second-line regimen, but median overall survival remained below seven months, underscoring the persistent unmet need [10].

The TRITICC-2 trial was designed to address this gap by evaluating a biologically rational combination therapy in a well-defined second-line population. A single-arm phase II design was intentionally selected to enable efficient signal detection in a disease setting where no universally accepted global standard exists and where randomized trials are challenging due to patient heterogeneity and limited eligibility for second-line treatment [8]. In this context, progression-free survival was chosen as the primary endpoint, representing a clinically meaningful measure of disease control and allowing comparison with historical benchmarks from prior second-line studies.

The study population reflects routine clinical practice by focusing on patients with unresectable or metastatic cholangiocarcinoma or gallbladder carcinoma who have progressed on gemcitabine-based therapy and maintain adequate performance status. Importantly, patients with actionable genomic alterations are only included if no approved targeted therapy is available or if such therapies are declined, acknowledging the increasing relevance of precision oncology approaches in BTC [16, 17]. However, as these alterations apply only to a subset of patients, the majority remain dependent on cytotoxic treatment strategies, reinforcing the clinical relevance of the present study.

The combination of trifluridine/tipiracil (FTD/TPI) and nanoliposomal irinotecan (nal-IRI) is supported by complementary mechanisms of action and emerging clinical evidence. FTD/TPI has demonstrated survival benefit in refractory gastrointestinal malignancies, including metastatic colorectal and gastric cancer [21, 22]. Irinotecan-based regimens have shown activity in BTC, particularly in combination with fluoropyrimidines [11], and the liposomal formulation nal-IRI has been associated with improved pharmacokinetics and clinical efficacy in pancreatobiliary malignancies. In the NIFTY trial, nal-IRI combined with fluorouracil and leucovorin significantly improved progression-free and overall survival in patients with previously treated BTC [12, 27], although results from the NALIRICC study were less pronounced [13]. These data collectively support further investigation of optimized irinotecan-based combinations.

TRITICC-2 extends this therapeutic concept by combining nal-IRI with FTD/TPI, a nucleoside analogue with a distinct mechanism of action that may overcome resistance to conventional fluoropyrimidines [20]. Preclinical and early-phase clinical studies suggest potential synergistic effects between FTD/TPI and irinotecan, possibly mediated through enhanced DNA damage and incorporation of trifluridine into DNA [23]. The use of an oral fluoropyrimidine backbone may further improve treatment feasibility in a patient population often limited by cumulative toxicity and performance status.

A key strength of TRITICC-2 is the integration of a comprehensive translational research program, including serial liquid biopsies, circulating RNA profiling, cytokine analyses, and microbiome assessment. Given the biological heterogeneity of BTC, such approaches are essential to identify molecular determinants of response and resistance and may facilitate the development of predictive biomarkers for future treatment strategies [2]. Previous studies have highlighted the relevance of genomic alterations and tumor biology in shaping therapeutic outcomes, but prospective biomarker-driven data in the second-line setting remain limited.

In summary, TRITICC-2 is designed to generate prospective evidence for a novel, biologically motivated combination therapy in a clinically relevant second-line BTC population. By combining efficacy, safety, and translational endpoints, the study aims not only to evaluate clinical activity but also to advance the understanding of treatment response in this challenging disease. The results may help inform future therapeutic strategies and contribute to the optimization of systemic treatment in advanced biliary tract cancer.

## Supplementary information

Below is the link to the electronic supplementary material.

Supplementary Material 1

## Data Availability

No datasets were generated or analysed during the current study.

## Declarations

### Where the trial protocol and statistical analysis plan can be accessed

The trial protocol is available on CTIS public portal (https://euclinicaltrials.eu/search-for-clinical-trials/?lang=en). The statistical analysis plan is available on request.

### Plans to communicate trial results to participants, healthcare professionals, the public, and other relevant groups (e.g., reporting in trial registry, plain language summary, publication)

Trial results will be made publicly available on CTIS public portal within one year of the study`s completion, including a lay person summary. Moreover, trial results, irrespective of positive or negative outcome, will be published by the CI in a peer reviewed journal and will be presented at German or international congresses. Any publication will take account of the ‘Uniform requirements for manuscripts submitted to biomedical journals (International Committee of Medical Journal Editors’ (ICMJE).

### Explanation of any interim analyses and stopping guidelines, including who will have access to these interim results and make the final decision to terminate the trial

No interim analyses are planned.

### Frequency and procedures for monitoring trial conduct

As part of a risk-based monitoring strategy, all sites will be visited on-site after first restaging visit of the first enrolled participant. Further visits with be scheduled according to e.g. number of enrolled patients, protocol deviations and findings from central monitoring activities. All sites will be visited on-site at least once a year. Monitoring activities on-site focuses on the trial data and processes that are critical to study integrity. Further details can be found in the monitoring plan. The on-site monitoring visits will be combined with central monitoring activities performed by data managers and biostatisticians. Further details can be found in the centralized monitoring plan.

## List of abbreviations

16S RNA: 16S ribonucleic acid
5-FU: 5-Fluorouracil
5-HT3: 5-hydroxytryptamine
AE: Adverse event
ALT: Alanine aminotransferase
AMG: German Medicine Act
ANC: Absolute neutrophil count
APRIL: A proliferation-inducing ligand
AST: Aspartate aminotransferase
BID: Bis in die (twice daily)
BSA: Body surface area
CCA: Cholangiocellular carcinoma
CCL: CC-chemokine ligand
cfDNA: Cell-free deoxyribonucleic acid
CNS: Central nervous system
CPT-11: Irinotecan
CT: Computed tomography
CXCR: C-X-C chemokine receptor
DNA: Deoxyribonucleic acid
ECOG: Eastern Cooperative Oncology Group
eCRF: Electronic case reporting form
EORTC: European Organisation for Research and Treatment of Cancer
EORTC QLQ-C30: Questionnaire developed to assess the quality of life of cancer patients
EQ-5D-5L: Questionnaire developed to assess the quality of life of cancer patients
EQ-5D-5L: European Quality of Life 5 Dimensions 5 Level Version
EU: European Union
EUCT: European Union Clinical Trials registration number
FACS: Fluorescence-activated cell sorting
FOLFIRI: 5-Fluorouracil, Leucovorin, Irinotecan
FOLFOX: 5-Fluorouracil, Leucovorin, Oxaliplatin
FTD/TPI: Trifluridine/Tipiracil
FUFIRI: Irinotecan, 5-fluorouracil, leucovorin
GCP: Good Clinical Practice
G-CSF: Granulocyte colony-stimulating factor
HR-QoL: Health-related quality of life
ICH: International Council for Harmonisation of Technical Requirements for Pharmaceuticals for Human Use
IDH1,2: Isocitrate dehydrogenase 1,2
IncRNA: Long non-coding ribonucleic acid
INR: International normalized ratio
KKSD: Coordination Center for Clinical Trials Düsseldorf
mCRC: Metastatic colorectal cancer
miRNA: Micro ribonucleic acid
MRI: Magnetic resonance imaging
NCI-CTCAE: National Cancer Institute Common Toxicity Criteria for Adverse Events
NCT: Clinical trials.gov registration number
OS: Overall survival
PFS: Progression free survival
PTT: Partial thromboplastin time
QoL: Quality of Life
qPCR: quantitative polymerase chain reaction
RECIST: Response Evaluation Criteria in Solid Tumours
RFA: Radiofrequency ablation
SADR: Serious adverse drug reaction
(S)AE: (Serious) adverse event
SGOT: Serum glutamic-oxaloacetic transaminase
SGPT: Serum glutamic pyruvic transaminase
SIRT: Selective intra-arterial radiotherapy
SMAD4: Mothers against decapentaplegic homolog 4
SmPC: Summary of Product Characteristics
suPAR: Soluble urokinase plasminogen activator receptor
TACE: Transarterial chemoembolization
TRAIL: Tumor necrosis factor-related apoptosis-inducing ligand
TRITICC: Efficacy and safety of Trifluridine/Tipiracil in combination with Irinotecan as a second-line therapy in patients with cholangiocarcinoma
TWEAK: Tumor necrosis factor-like weak inducer of apoptosis
ULN: Upper limit of normal
VAS: Visual analogue scale
